# Mechanisms of Programmed Cell Death in Sodium Iodate-Driven Retinal Degeneration and the Role of DJ-1

**DOI:** 10.3390/ijms27062541

**Published:** 2026-03-10

**Authors:** Mala Upadhyay, Caroline Milliner, Vera L. Bonilha

**Affiliations:** 1 Department of Ophthalmic Research, Cole Eye Institute, Cleveland Clinic, 9500 Euclid Avenue, Cleveland, OH 44195, USA; upadhym@ccf.org (M.U.); millinc2@ccf.org (C.M.); 2 Department of Ophthalmology, Cleveland Clinic Lerner College of Medicine, Case Western Reserve University, Cleveland, OH 44195, USA

**Keywords:** DJ-1, RPE, retina, apoptosis, necroptosis, sodium iodate

## Abstract

Oxidative stress-induced RPE cell death is a major cause of AMD pathogenesis. However, the exact modes of oxidative stress-driven retinal death remain elusive. To address this knowledge gap, we investigated the role of DJ-1, an antioxidant protein we previously characterized in the retina, in cell death regulation. Specifically, we analyzed cell death pathways in the retinas of DJ-1 knockout (KO) mice, with or without sodium iodate (NaIO_3_) injection. We quantified MAPK signaling protein activation by Western blot. The distribution of the cell death executioners, activated caspase 3, and pMLKL, was investigated. The effects of caspase and necroptosis inhibitors in mice previously injected with NaIO_3_ were determined. Significant increases in JNK1/2 activation and FOXO1 levels were detected in RPE lysates when DJ-1 KO mice were injected with 10 mg/kg NaIO_3_. The immunoreactivity of active caspase-3 and pMLKL was stronger in the retinas of DJ-1 KO compared with C57BL mice. These immunoreactivities further increased in the degenerating outer retina post NaIO_3_ injection and were stronger in the retina of DJ-1 KO compared with C57BL mice at both doses of NaIO_3_. ZVAD treatment rescued retinal degeneration to varying degrees in DJ-1 KO mice. However, necrostatin (Nec-1) alleviated retinal degeneration in both DJ-1 KO and C57BL mice, suggesting that apoptosis is a major cell death modality in the absence of DJ-1. Overall, oxidative stress-induced RPE and retinal cell death involve activation of both apoptosis and necroptosis in the absence of DJ-1.

## 1. Introduction

Age-related macular degeneration (AMD) is the third leading cause of blindness in aging adults in developed countries [1]. AMD can be classified into three main clinical states: early, intermediate, and advanced. In the early phase of AMD, there is chronic low-level inflammation, deposition of drusen beneath the retinal pigment epithelium’s (RPE) Bruch’s membrane, followed by patchy cell death and subsequent choriocapillaris and photoreceptor cell death in the macula region of the retina, with inconspicuous vision loss [2,3,4]. The complexity of AMD pathophysiology is attributed to several risk factors, inflammation and oxidative stress being one of the major drivers of RPE and photoreceptor cell death and AMD development. Previous studies have suggested apoptosis as a major regulated cell death pathway in this pathology [5,6]. In contrast, more recent studies have indicated the involvement of multiple regulated cell death pathways in RPE and the underlying photoreceptor due to oxidative stress [7,8,9]. No consensus has yet been reached to define the mechanisms of RPE and photoreceptor cell loss, and this remains an open question in the quest to find new modalities for the treatment of AMD.

The RPE possesses a powerful antioxidant system that combats the high levels of oxidative stress induced by acute light exposure and the retina’s high metabolic rate. However, in diseases such as AMD, overproduction of free radicals leads to oxidative damage to biomolecules and organelles, such as mitochondria. Nonetheless, the detailed molecular mechanisms underlying the initiation of the pathophysiological changes in AMD remain largely unknown. Thus, in this study, we used sodium iodate (NaIO_3_), a potent oxidizing agent that preferentially affects RPE when systemically injected [10,11,12]. The NaIO_3_ model of degeneration recapitulates AMD-like patchy RPE loss [11,12,13] and allows longitudinal assessment of retinal structure in a dose-dependent manner across several mammalian species and in cell lines [14,15,16,17,18].

The *PARK7* gene encodes DJ-1, a multifunctional protein that protects neurons from oxidative stress [19,20]. Previous studies from several groups and ours have established that DJ-1 levels increase [21,22,23] and that DJ-1’s cysteine residues can be post-translationally modified to alter DJ-1’s activity in response to increased oxidative stress, helping fight it [24,25]. Indeed, our previous work observed increased amounts of sulfonic oxidized DJ-1, the overoxidized, inactive form of the protein in the RPE of AMD donors [22]. Therefore, we continued our studies analyzing retinal oxidative stress using the DJ-1 knockout (KO) mouse model. In the absence of DJ-1, aging mice (18 months old) show RPE and retinal degeneration, with increased accumulation of oxidative stress markers, such as protein carbonylation and inducible nitric oxide synthase expression, suggesting that DJ-1 is essential for protection against oxidative stress in the retina with aging [26]. Using the NaIO_3_ model and DJ-1 KO mice, we have previously shown that a single tail vein injection of NaIO_3_ accelerated outer retinal atrophy [17]. Moreover, we also demonstrated that a low dose of NaIO_3_ (10 mg/kg) is sufficient to induce RPE and retinal degeneration in DJ-1 KO mice but not in controls (C57BL/6J), independent of age [27].

To further understand how RPE and retina degeneration in DJ-1 KO mice respond to increased oxidative stress, here we have induced oxidative stress using 10 mg/kg (low dose) and 20 mg/kg (high dose) of NaIO_3_ in 3-month-old mice to characterize the cell death pathways in the retina and RPE. We initially investigated the mammalian family of mitogen-activated protein kinases (MAPKs), including extracellular signal-regulated kinase (ERK), P38, and c-Jun N-terminal kinase (JNK), in DJ-1 KO and C57BL mice. Then, we examined the executioners of apoptosis and necroptosis and the inhibition of these pathways in both mouse groups. Specifically, we aimed to understand the mechanistic role of DJ-1 in retinal and RPE cell death and pathology, and to elucidate the molecular underpinnings of dry AMD further.

## 2. Results

### 2.1. Cell Death and Survival Are One of the Top Modulated Molecular Functions in DJ-1 KO Mice

Our previous data showed that loss of DJ-1 renders the RPE antioxidant machinery unable to combat and neutralize low-level oxidative stress in both young (3 months) and old (15 months) mice. Specifically, loss of DJ-1 KO results in upregulation of antioxidant genes in young DJ-1 KO RPE and downregulation of these in the retina, adversely affecting antioxidant machinery and resulting in the increased deposition of advanced glycation end products (AGEs) and neutral lipid beneath RPE [27].

**Figure 1:**
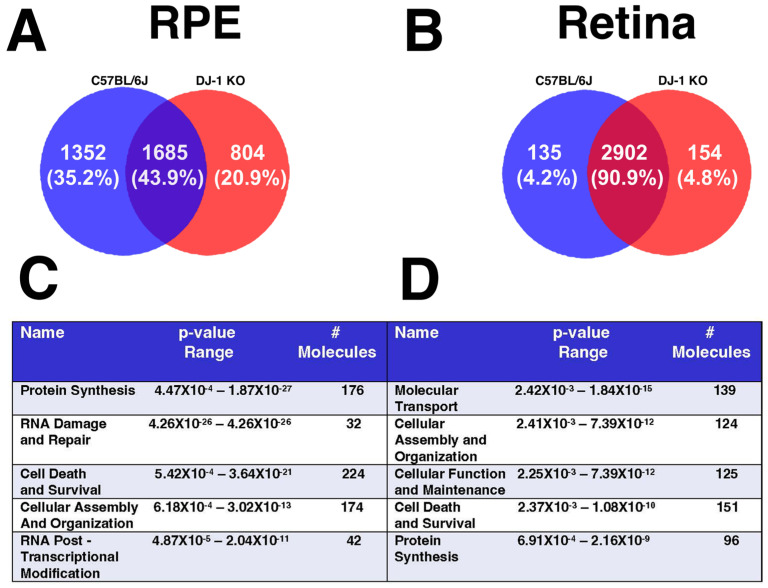
Baseline proteomics changes in RPE and retina of the DJ-1 KO mice. (**A**) Venn diagram of overlapping and unique proteins in the RPE and (**B**) retina of the C57BL/6J and DJ-1 KO mice. (**C**) Ingenuity pathway analysis (IPA) of the top five molecular and cellular functions altered in the RPE and (**D**) retina of the DJ-1 KO mice; *n* = 4.

To further explore the reasons behind the preferential RPE and retinal degeneration in DJ-1 KO mice, we took an unbiased approach and performed proteomics analyses of RPE and retina from 3-month-old C57L/6J and DJ-1 KO mice at baseline conditions and after exposure to low-level oxidative stress. The details of proteomics processing and data analysis can be accessed at [27] and https://doi.org/10.5061/dryad.xpnvx0kb6, respectively. The LC-MS/MS data quantification was performed with the label-free quantitation method in the MaxQuant program [28]. At baseline conditions, a total of 1352 proteins were uniquely identified in C57BL RPE, whereas 804 proteins were unique in DJ-1 KO mice. In the retina, 135 proteins were unique to C57BL, whereas 154 proteins were unique to DJ-1 KO (Figure 1A,B). It was intriguing to observe that unique proteins in the RPE of C57BL and DJ-1 KO were approximately 8–9 times higher than the number of unique proteins in the retina of controls and DJ-1 KO mice. This observation suggests that loss of DJ-1 can have profound effects on proteomic topography in RPE alone. Further analysis of the list of identified proteins using the ingenuity pathway analysis (IPA) tool was then performed, and determined “Cell Death and Survival” as one of the top five molecular and cellular functions modulated in both the RPE and retina of DJ-1 KO mice at baseline conditions (Figure 1C,D). A detailed list of the 224 proteins altered in the RPE and the 151 proteins altered in the retina in the DJ-1 KO, along with the diseases and functions annotated to them, including those involved in cell death and survival, is also provided (Supplementary Tables S1 and S2).

### 2.2. MAPK Signaling Is Modulated in RPE with a 10 mg/kg Dose of NaIO_3_ (Low-Level) Stress

**Figure 2:**
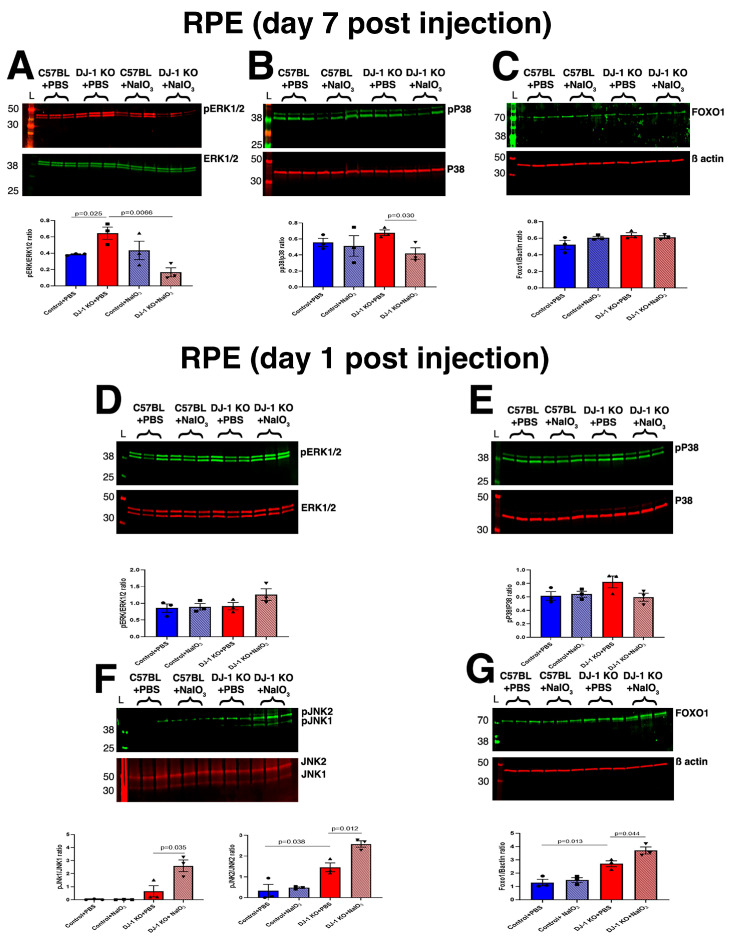
MAPK signaling in RPE of the DJ-1 KO mice after NaIO_3_ injection. Representative Western blot images and their quantification of (**A**) pERk1/2 and ERK1/2, (**B**) pP38 and P38, and (**C**) FOXO1 at day 7 post PBS and NaIO_3_ injection of C57BL and DJ-1 KO mice. Representative Western blot images and their quantification of (**D**) pERk1/2 and ERK1/2, (**E**) pP38 and P38, (**F**) pJNK1/2 and JNK1/2, and (**G**) FOXO1 at day 1 post PBS and NaIO_3_ injection of C57BL and DJ-1 KO mice; data are expressed as mean ± SEM. Significance values are indicated on the graphs, Student’s *t*-test, *n* = 3.

The literature suggests involvement of the the MAPK signaling pathway in oxidative stress-mediated RPE degeneration [29,30,31]. Moreover, multi-locus enrichment analysis and functional pathway network analyses using changes in gene expression of single genes between AMD and normal eyes implicate MAPKs in AMD pathogenesis [32,33]. Specifically, ERk1/2, P38 and JNK1/2 are the MAP kinases that are activated and which translocate to the nucleus to modulate the expression of their target genes involved in survival, proliferation, and cell death. To test the role of DJ-1 expression and oxidative stress in MAPK signaling, we individually quantified the levels of pERk1/2, pP38, and pJNK1/2 using Western blots in the RPE and retinas of both C57BL/6J and DJ-1 KO mice after injection with PBS or NaIO_3_. At day 7 post-injection, there was a significant increase in pERK1/2 levels in the RPE of DJ-1 KO injected with PBS, indicating activation of pathways involved in cell survival or apoptotic cell death; pP38 levels were not changed. With the injection of 10 mg/kg NaIO_3_, RPE cells degenerated and died, and both pERK1/2 and pP38 levels significantly decreased in the RPE of DJ-1KO compared with PBS-injected DJ-1 KO mice, suggesting that pERK1/2 and pP38 might be associated with cell death. We did not detect changes in these phosphorylated proteins in the RPE of C57BL mice injected with NaIO_3_ (Figure 2A,B). We also quantified the levels of pJNK1/2 and JNK1/2 in the RPE for the same groups after injection of 10 mg/kg NaIO_3_, but the levels of these proteins were undetectable in the same amount of lysate probed. Several studies have reported JNK1/2 signaling and forkhead box protein O1 (FOXO1), a downstream target of JNK1/2 signaling in the oxidative stress-induced cell death axis [34,35,36]. To further probe JNK1/2 involvement in RPE cell death, we quantified FOXO1 levels but did not detect changes in any of the mouse groups analyzed (Figure 2C).

As we could not quantify pJNK1/2 and JNK1/2 in the RPE of both the DJ-1 KO and C57BL mice, and because JNKs are rapidly activated by phosphorylation in response to diverse stressors such as oxidative stress, we proceeded to collect RPE lysates 1 day after injection with PBS or 10 mg/kg of NaIO_3_, before the RPE degenerated and died in the DJ-1 KO (Figure 2D–G). This approach was also based on our previous observations that RPE begins to show signs of stress and starts to degenerate as early as day 1 post-NaIO_3_ injection [17]. To our surprise, at day 1 post-injection, we detected increased levels of pJNK1/2 in the RPE of DJ-1 KO after injection with PBS as well as with 10 mg/kg NaIO_3_. The levels of pJNK1and pJNK2 were significantly increased in the RPE of NaIO_3_-injected DJ-1 KO mice when compared with PBS-injected DJ-1 KO mice, whereas the levels of pERK1/2 and pP38 remained unchanged in all groups (Figure 2D–F). The FOXO1 levels were also increased in the RPE 1 day after injection with PBS and 10 mg/kg NaIO_3_ (Figure 2G). Overall, our data suggest that pJNK1/2 is activated in the RPE of DJ-1 KO mice as early as day 1 post NaIO_3_-mediated oxidative stress and might initiate cell death signaling, and that, in parallel, FOXO1 levels also increase to cope with the deleterious effects of oxidative stress and support survival. As the RPE cells start to degenerate by day 7, the levels of pERK1/2 and pP38 decrease further in DJ-1 KO mice, as the RPE protective stress response mechanism fails, leading to increased cell damage and cell death.

**Figure 3:**
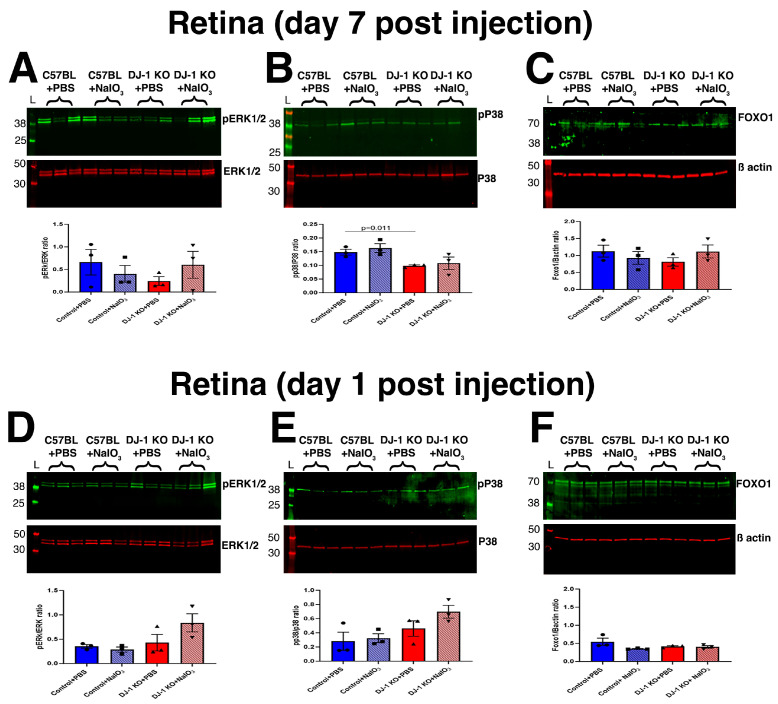
MAPK signaling in the retina of the DJ-1 KO mice after NaIO_3_ injection. Representative Western blot images and their quantification of (**A**) pERk1/2 and ERK1/2, (**B**) pP38 and P38, and (**C**) FOXO1 at day 7 post PBS and NaIO_3_ injection of C57BL and DJ-1 KO mice. Representative Western blot images and their quantification of (**D**) pERk1/2, (**E**) ERK1/2 and pP38, P38, and (**F**) FOXO1 at day 1 post PBS and NaIO_3_ injection of C57BL and DJ-1 KO mice; data are expressed as mean ± SEM. Significance values are indicated on the graphs, Student’s *t*-test, *n* = 3.

Next, we performed a similar analysis in retina lysates. At day 7 post-injection, no significant changes in the levels of pERK1/2 (Figure 3A,C) and FOXO1 between groups were observed. However, there was a significant decrease in pP38 levels after PBS injection in the retina of DJ-1 KO compared with C57BL mice (Figure 3B). We did not detect significant changes after injection of 10 mg/kg NaIO_3_ in any of the retinas of the mice analyzed. At day 1 post NaIO_3_ injection, a trend of increase was observed for pERK1/2 (Figure 3D) and pP38 (Figure 3E) in the retinas of DJ-1 KO mice injected with NaIO_3_ when compared with mice injected with PBS. The FOXO1 levels remained comparable across all the mouse groups analyzed (Figure 3F). We were unable to detect JNK in retinal lysates. Altogether, our data suggest that the most significant failure in survival signaling occurs in the RPE of DJ-1 KO mice after NaIO_3_ injection. In contrast, the retinal cells of DJ-1 KO mice showed lower levels of these proteins, with only a trend toward activation at day 1 after NaIO_3_ injection, potentially driven by the RPE changes.

To further understand the cell death signaling in DJ-1 KO mice, we also investigated the expression of death domain-associated protein (Daxx), as it has been shown to activate apoptosis signal-regulating kinase 1 (ASK1), the upstream JNK kinase, and an intermediary messenger for pro-apoptotic (cell death) signals, which activate JNK and p38 pathways in response to stress [37,38,39,40]. To do so, we performed immunohistochemistry to detect the expression of Daxx protein 7 days post-injection (Supplementary Figure S1). The retinas of C57BL mice injected with PBS displayed low levels of labeling, with minor labeling of the RPE, photoreceptor inner segments (IS) and the ganglion cell layer (GCL) (Supplementary Figure S1A, arrowheads). However, we detected increased diffused Daxx in RPE, photoreceptor IS, in the inner and outer plexiform layers (IPL and OPL), the inner nuclei layer (INL), and the GCL in C57BL injected with 10 mg/kg NaIO_3_ (Supplementary Figure S1B). In DJ-1 KO mice injected with PBS (Supplementary Figure S1D) we observed a similar pattern of Daxx distribution with the added distribution of a thin continuous line at the base of the ONL (Supplementary Figure S1D, double arrowheads). In C57BL injected with 20 mg/kg NaIO_3_ (Supplementary Figure S1C) and in DJ-1 KO mice injected with 10 mg/kg NaIO_3_ (Supplementary Figure S1E), the Daxx labeling was observed in the same areas, but at lower levels and more defined, punctated in the inner layers of the retina, namely in the GCL, IPL and INL of the retina and along the degenerating photoreceptor IS. In DJ-1 KO mice injected with 10 mg/kg NaIO_3_, a thin continuous line at the base of the ONL was also very evident (Supplementary Figure S1E, double arrowheads). This pattern persists in the highly degenerated retinas of the DJ-1 KO mice injected with 20 mg/kg NaIO_3_ (Supplementary Figure S1F). Daxx acts as an adapter protein, linking cell-surface death receptors (such as the Fas receptor) to the JNK signaling pathway. Thus, these data, in conjunction with the increased JNK1/2 and FOXO1 activation detected in RPE lysates from DJ-1 KO mice injected with NaIO_3_, suggests that several retinal cells are undergoing a significant stress response, one that leads to programmed cell death.

### 2.3. Apoptosis Inhibition Rescues RPE and Retinal Degeneration in DJ-1 KO Mice Exposed to Oxidative Stress

**Figure 4:**
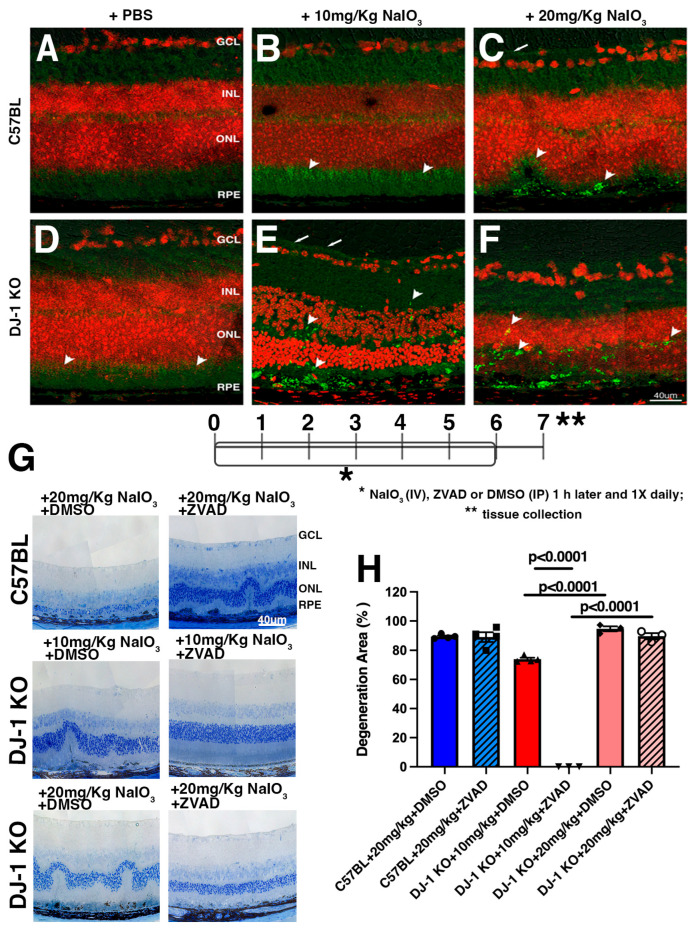
Caspase 3 activation in the retinas of mice after NaIO3 injection. (**A**–**F**) Representative images displaying acasp3 (green) immunoreactivity in RPE and retina of DJ-1 KO and C57BL mice injected with NaIO_3_; red: TO-PRO-3; bar = 40 μm. (**G**) Experimental flow, representative images of toluidine blue-stained retinas, bar = 40 μm. (**H**) Quantification of degenerated area from DJ-1 KO and C57BL mice injected with NaIO_3_ followed by DMSO and ZVAD; data are expressed as mean ± SEM. Significance values are indicated on the graph, Two-way Anova, *n* = 3–7.

Previous studies have suggested that apoptosis, necroptosis, autophagy, and other regulated cell death pathways may be involved in RPE, and in retinal cell death induced by NaIO_3_ oxidative stress [41,42,43,44]. To further understand the mode of cell death in response to NaIO_3_, we first investigated activation of apoptosis by labeling the retinal cryosections with active caspase 3 (acasp3), as it is considered the executioner caspase in this pathway by cutting up key proteins, leading to cell dismantling, DNA fragmentation, and the formation of apoptotic bodies [45]. The retinas of C57BL mice injected with PBS and probed with an acasp3 antibody displayed low levels of labeling (Figure 4A). The retinas of DJ-1 KO mice displayed increased expression of acasp3 in the IPL and in the photoreceptor IS when PBS was injected (Figure 4D, arrowheads), and the retinas of C57BL mice exposed to low levels of oxidative stress (10 mg/kg NaIO_3_) displayed a similar acasp3 labeling, but with increased levels (Figure 4B, arrowheads). Injection of the DJ-1 KO mice with low-level oxidative stress resulted in retinal degeneration, with acasp3 labeling observed as a thin continuous line in the GCL (Figure 4E, arrows) and as green dots interspersed in the INL, OPL, photoreceptor IS, RPE, and choroid layers (Figure 4E, arrowheads). We previously reported that both C57BL and DJ-1 KO mice injected with 20 mg/kg NaIO_3_ (higher dose) developed retinal degeneration [27]. Exposure to higher levels of oxidative stress in C57BL mice resulted mostly in the concentrated distribution of acasp3 in the degenerating RPE and photoreceptor layer (Figure 4C, arrowheads) but also in a thin continuous line in the GCL (Figure 4C, arrow), whereas acasp3 was expressed in degenerating RPE, the photoreceptors, ONL, and INL in the degenerating retina of DJ-1 KO mice (Figure 4F, arrowheads). These observations suggest that apoptosis is mostly triggered in the outer retina in C57BL mice. However, in the more sensitive DJ-1 KO mice, it extends into the inner retina in response to NaIO_3_.

We further confirmed our previous observation of caspase-3 activation and retinal degeneration by treating mice with ZVAD-fmk, a pan-caspase inhibitor previously shown to partially block cell death in the light-damage model of retinal degeneration [46]. Our experimental workflow included an initial tail vein injection of NaIO_3_, followed by an intraperitoneal injection of ZVAD-fmk or DMSO 1 h later and once a day till day 6. On day 7 after injections, samples were collected and processed for histological analysis. As described above, we did not include the C57BL mice injected with 10 mg/kg NaIO_3_ because no degeneration was observed at this dose (Figure 4B) and in our previously reported data [27]. Our histological observations around the optic nerve head indicated that, following exposure of C57BL mice to 20 mg/kg NaIO_3_, retinal degeneration was observed independently of ZVAD-fmk treatment (Figure 4G). However, following exposure of DJ-1 KO mice to NaIO_3_, retinal degeneration was completely (after injection of 10 mg/kg NaIO_3_) or partially blocked (after injection of 20 mg/kg NaIO_3_) when mice were also treated with ZVAD-fmk. Quantification of the area of RPE degeneration in the retinal sections of DJ-1 KO mice injected with 10 and 20 mg/kg NaIO_3_ + DMSO detected ~74% and 95% degeneration, respectively (Figure 4H). In C57BL mice injected with 20 mg/kg NaIO_3_ + DMSO, ~89% retinal degeneration was detected. In DJ-1 KO mice injected with 10 mg/kg NaIO_3_ + ZVAD-fmk, the retinal degeneration was totally rescued, whereas ~89% retinal degeneration was observed in DJ-1 KO mice injected with 20 mg/kg NaIO_3_ + ZVAD-fmk. In C57BL mice injected with 20 mg/kg NaIO_3_ + ZVAD-fmk, no significant decrease in retinal degeneration was observed (Figure 4H). These observations suggest a strong protective role for DJ-1 against apoptosis at low levels of oxidative stress.

### 2.4. Necroptosis Inhibition Attenuates RPE and Retinal Degeneration in Mice Exposed to Oxidative Stress

**Figure 5:**
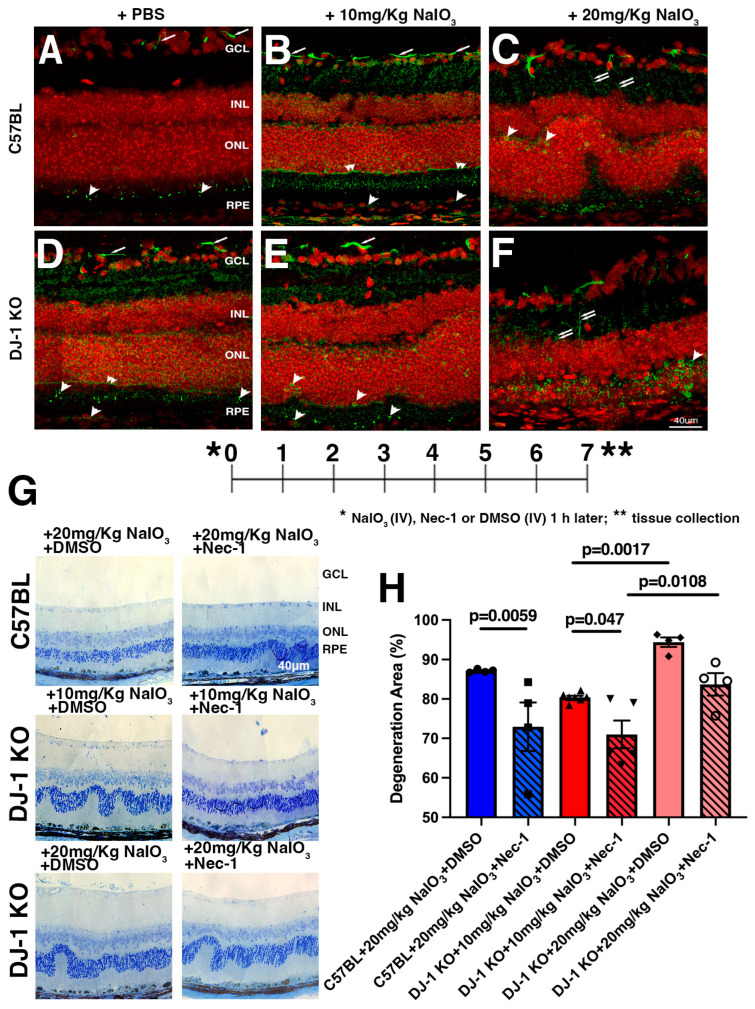
MLKL activation in the retinas of mice after NaIO3 injection. (**A**–**F**) Representative images displaying pMLKL (green) immunoreactivity in RPE and retina of DJ-1 KO and C57BL mice injected with NaIO_3_; red: TO-PRO-3; bar = 40 μm. (**G**) Experimental flow, representative images of toluidine blue-stained retinas; bar = 40 μm. (**H**) Quantification of degenerated area from DJ-1 KO and C57BL mice injected with NaIO_3_ followed by DMSO and Nec-1 treatment; data are expressed as mean ± SEM. Significance values are indicated on the graph, Two-way Anova, *n* = 4–7.

To further detail the cell death pathway triggered by NaIO_3_ exposure, we next investigated the phosphorylation of mixed lineage kinase domain-like pseudo kinase (MLKL), as phosphorylated MLKL (pMLKL) forms pores in the plasma membrane and causes necroptosis [45]. The retinas of C57BL mice injected with PBS and probed with a pMLKL antibody displayed strong labeling in the GCL (Figure 5A, arrows) and dotted labeling of the photoreceptor IS (Figure 5A, arrowheads). The retinas of DJ-1 KO mice injected with PBS displayed a similar pattern of pMLKL distribution, with the additional labeling of a thin continuous line at the base of the ONL (Figure 5D, double arrowheads) and the RPE (Figure 5D, arrowheads). The retinas of C57BL mice exposed to a low level of oxidative stress (10 mg/kg NaIO_3_) displayed a similar pMLKL labeling to that observed in DJ-1 KO mice injected with PBS, with increased levels in the GCL (Figure 5B, arrows), dotted labeling of the photoreceptor IS (Figure 5B, arrowheads), and a continuous line at the base of the ONL (Figure 5B, double arrowheads). Injection of the DJ-1 KO mice with low-level oxidative stress resulted in retinal degeneration, with pMLKL labeling becoming discontinuous in the GCL (Figure 5E, arrows) and less abundant in the photoreceptor IS, as well as resulting in the appearance of labeling around several nuclei interspersed in the ONL, and in concentrated labeling spots in the ONL and OPL (Figure 5E, arrowheads). Exposure to higher levels of oxidative stress in C57BL mice resulted in retinal degeneration, with pMLKL labeling becoming discontinuous in the GCL; labeling in Müller cells, with distribution advancing towards the INL along cell processes (Figure 5C, double arrows) and becoming less abundant in the photoreceptor IS; and the appearance of labeling around several nuclei interspersed in the ONL (Figure 5C, arrowheads). In the retinas of DJ-1 KO injected with 20 mg/kg NaIO_3_, pMLKL was decreased in GCL, increased in Müller cell processes (Figure 5F, double arrows), and increased in the ONL, OPL, and choroid (Figure 5F, arrowheads). These observations suggest that necroptosis is initiated more uniformly in the inner and outer layers of the retina in mice, and that it becomes localized to the outer retina as it degenerates in response to higher doses of NaIO_3_.

We further confirmed our previous observation of MLKL activation and retinal degeneration by treating mice with Nec-1, an inhibitor of RIP1 Kinase and the necroptosis pathway that has been previously shown to partially block cell death in several animal models exposed to NaIO_3_ [47,48,49]. Our experimental workflow included an initial tail vein injection of NaIO_3_, followed by a single tail vein injection of Nec-1 or DMSO 1 h later. On day 7 after injections, samples were collected and processed for histological analysis. As explained above, we did not include the C57BL mice injected with 10 mg/kg in these experiments because no degeneration was observed at this dose (Figure 4B and Figure 5B). Our histological observations around the optic nerve head indicated that following exposure of C57BL to 20 mg/kg NaIO_3_, retinal degeneration was partially inhibited by Nec-1 treatment (Figure 5G). Quantification of the area of RPE degeneration in the retinal sections of DJ-1 KO mice injected with 10 and 20 mg/kg NaIO_3_ + DMSO was ~78% and 95%, respectively, while, in C57BL mice injected with 20 mg/kg NaIO_3_ + DMSO, ~87% retinal degeneration was observed. In DJ-1 KO mice injected with Nec-1, the retinal degeneration was ~71% in mice injected with 10 mg/kg NaIO_3_ and ~84% in mice injected with 20 mg/kg NaIO_3_, which implies that Nec-1 displays a partial protective effect at both NaIO_3_ concentrations tested. In C57BL mice injected with 20 mg/kg NaIO_3_ + Nec-1, ~73% retinal degeneration was detected (Figure 5H). These observations suggest a role for necroptosis in NaIO_3_-induced retinal cell death. They also emphasize the increased susceptibility of DJ-1 KO mice to low-level oxidative stress, and the mirrored expression of cell death executors in C57BL mice compared with DJ-1 KO mice under increased levels of oxidative stress.

## 3. Discussion

The powerful antioxidant system of the RPE, which combats high levels of oxidative stress induced by acute light exposure and the retina’s high metabolic rate, is overwhelmed in diseases such as AMD, where we observe overproduction of free radicals. This disruption of the redox balance contributes to RPE and photoreceptor death in many retinal degenerative diseases, including AMD [50,51,52]. DJ-1, a protein first described as a Parkinson’s disease-related protein, has cytoprotective effects and protects cells from oxidative stress-mediated cell death [53,54]. Here, we examined the cell death mechanisms in the retina and RPE and found that oxidative stress-induced RPE and retinal cell death involve activation of both apoptosis and necroptosis in the absence of DJ-1.

In recent years, several studies have suggested that apoptosis and other regulated cell death pathways, including necroptosis [48,55,56], ferroptosis [9,48,49,57,58,59], pyroptosis [45,60,61,62,63,64], and cuproptosis [65], are involved in RPE and retinal cell death in AMD. Despite these advances, the exact mechanism and events leading to RPE and retinal cell death in AMD pathogenesis remain unclear. Therefore, in this study, we extended our previous observations by exposing C57BL and DJ-1 KO mice to increasing concentrations of the oxidizing agent NaIO_3_. This model has been used in many in vivo and in vitro studies to induce patchy RPE loss, secondary photoreceptor degeneration, and underlying choriocapillaris death, resembling human AMD pathology, due to its flexibility in modeling time- and dose-dependent degeneration observed in diverse animal models [14,45,66,67,68].

With the knowledge from our previous work, which detected increased amounts of sulfonic oxidized DJ-1, the overoxidized, inactive form of the protein in the RPE of AMD donors, we re-analyzed proteomics data from retina and RPE of DJ-1 KO and C57BL mice. Our analysis identified cell death pathways as one of the top five molecular and cellular functions modulated in the RPE and retina of DJ-1 KO compared with C57BL (Figure 1). Furthermore, proteomics analyses indicated that the RPE and retina in DJ-1 KO are already physiologically stressed, and that factors associated with cell death are highly expressed in them. We then quantified MAPK signaling protein levels (Figure 2 and Figure 3) because this signaling pathway has been reported to be associated with the survival and death of many cell types and tissues, including the retina and RPE [30,69,70]. Our results agree with previous studies showing that DJ-1 can activate the ERK1/2 pathway [71,72]. We observed an increased level of pERK1/2 in the RPE of DJ-1 KO injected with PBS, corroborating our proteomics data that suggest that cell survival and death pathways are active in DJ-1 KO RPE and retina, which is also in accordance with a previous manuscript that reported that wild-type RPE displayed a fast biphasic phosphorylation of ERK1/2 while the Nrf2 KO mice only displays phosphorylation at early stages of stress [30].

It has also been shown that DJ-1 can activate the phosphatidylinositol-3-kinase (PI3K)/Akt pathway [73] to mediate cell survival and proliferation, and it can also attenuate cell death signaling by inhibiting ASK1 activation [74,75]. We observed increased levels of pJNK1/2 only in DJ-1 KO RPE at day 1 after NaIO_3_ injection. This result is in line with that of another study showing that pP38 and pJNK1/2 are activated by reactive oxygen species (ROS) induced by NaIO_3_ in the human RPE cell line ARPE-19 [76]. We aimed to further dissect the cell death pathway underlying increased stress in the absence of DJ-1. However, we were unable to detect ASK1, the JNK kinase activator, in the RPE lysates from the analyzed mice. We observed Daxx distribution in our mouse groups (Supplementary Figure S1). Our study found that diverse stresses clearly induce Daxx protein levels, an effect also observed in C57BL mice but not in DJ-1 KO retinas. Notably, a previous study demonstrated that Daxx localizes to the nuclei of cells [38]. Moreover, DJ-1 sequesters Daxx in the nucleus and prevents its cytoplasmic translocation, where it binds to and activates ASK1 [77]. We failed to detect Daxx distribution in the nuclei of retinal cells under any conditions, which may be related to the experimental performance 7 days after injection of NaIO_3_ and PBS.

Our findings also suggest that activation of pJNK1/2 in RPE is an early response to increased stress, leading to RPE cell death. Alternatively, pERK1/2 might be pro-survival in the DJ-1 KO mice, which is why, as RPE cells die when exposed to NaIO_3_, we see a decrease in pERK1/2. We did not observe significant alterations in MAPK signaling protein levels in the retina of any of the mouse groups analyzed (Figure 3). This could be related to exposure of mice to NaIO_3_, which preferentially affects the RPE, or it may indicate that cell death signaling triggered by oxidative stress initiates in the RPE. Further experiments are needed to better understand our observations.

Our proteomic analysis of the cell death pathways altered in the DJ-1 KO, focusing on the diseases and functions annotated within this pathway, highlights apoptosis and necrosis (Supplementary Tables S1 and S2). Moreover, to better understand the mode of cell death in response to NaIO_3_, we investigated the distribution of executioners of apoptosis (activated caspase 3, Figure 4) and necroptosis (pMLKL, Figure 5) in retinal cryosections. Our data show that both proteins are activated in response to NaIO_3_-mediated oxidative stress. Active caspase 3 was mainly observed in the IS layer of retina in DJ-1 KO mice injected with PBS, but it redistributed to the INL, ONL, and OPL after NaIO_3_ injection. This observation agrees with a previous study that reported a large number of apoptotic cells detected by TUNEL labeling in rat RPE cells and the ONL 7 days after treatment with NaIO_3_ [78] and with another study analyzing retro-orbital NaIO_3_ injection in C57BL mice [47]. In contrast, pMLKL was strongly localized to the GCL and photoreceptor IS, with faint labeling in the RPE of DJ-1 KO mice injected with PBS. Meanwhile NaIO_3_ injection resulted in pMLKL labeling becoming discontinuous in the GCL, less abundant in the photoreceptor IS, appearing around several nuclei interspersed in the ONL, and concentrated in spots in the ONL and OPL. The unique distribution of each cell death executor indicates that DJ-1 is differentially regulated across retinal cell types. Most importantly, our data strongly suggest that RPE and photoreceptors can die by both apoptosis and necroptosis. Our data are in line with the data of other studies, which suggest that more than one regulated cell death pathway can be responsible for the degeneration observed in microarray gene expression data from RPE-choroid isolated from AMD patients [33].

One limitation of our study is that we could not verify the exact mode of cell death involved in the RPE degeneration in both genotypes because of the extensive RPE degeneration in DJ-1 KO and in C57BL mice injected with 20 mg/kg NaIO_3_. Nevertheless, complete rescue of retinal degeneration after injection of the apoptosis inhibitor ZVAD-fmk suggests that apoptosis may be the main RPE degeneration cell death pathway in stressed DJ-1 KO mice and, therefore, that the presence of DJ-1 is strongly protective against apoptosis under physiological low-level oxidative stress (Figure 4G,H). We also determined that Nec-1 treatment partially rescued 7–8% of the degeneration at higher doses of NaIO_3_ in DJ-1 KO mice and around ~15% in C57BL mice. These observations contrast with another study, which showed that necroptosis was a major pathway associated with RPE cell death in response to NaIO_3_ [47]. This discrepancy in the data could be due to differences in the route, the concentration of the injected inhibitor, and the time point at which degeneration was scored in that study. Additional experiments testing different concentrations and treatment regimens might also improve protection against degeneration triggered by increased stress induced by NaIO_3_ exposure and provide novel treatments for oxidative stress-mediated cell death in the retina of AMD patients.

This report is the first to link DJ-1 with RPE and photoreceptor cell death via apoptosis and necroptosis. Our study suggests that, at low levels of oxidative stress, apoptosis can be the predominant mode of cell death in the retina and RPE. However, as oxidative stress increases and inflammation ensues, other cell death pathways, such as necroptosis, an inflammatory cell death pathway, can lead to retinal cell death. Furthermore, our observations can be used to compare with the actual AMD pathological events, in which the initial accumulation of ROS that is due to aging and a dysfunctional antioxidant response activates the cell death machinery in outer retinal cells, leading to apoptosis. As cells die by apoptosis and ROS persists chronically, it creates an adequate microenvironment for inflammation activation and, therefore, inflammation-associated regulated cell death pathways like necroptosis. Taking these specifics into consideration, it will be helpful to use a combination of apoptosis and necroptosis inhibitors to potentially inhibit RPE and retinal cell death in a high-throughput approach.

## 4. Materials and Methods

### 4.1. Mice

All animal procedures were performed in accordance with the Association for Research in Vision and Ophthalmology (ARVO) Statement for the Use of Animals in Ophthalmic and Vision Research and approved by the Institutional Animal Care and Use Committee (IACUC protocol number ARC2021-2191 and 00003449) at the Cleveland Clinic. DJ-1 KO mice have a deletion in exon2 and have been characterized earlier [79]. Mice were bred into C57BL/6J background and housed in individually ventilated cages (4/cage) in a 14 h light/10 h dark cycle and were provided regular chow and water ad libitum. Young adult mice (3-month-old) male and female were used in experiments, and age-matched C57Bl/6J mice were used as control. Retina of DJ-1 KO has been extensively characterized previously [17,26].

### 4.2. Sodium Iodate Degeneration Model

To induce degeneration, mice received a single tail vein injection of 10 and 20 mg/kg NaIO_3_ (71702, Sigma-Aldrich, St. Louis, MO, USA) of body weight made in PBS; the control group received PBS as previously described [17,27]. Mice were sacrificed on day 1 and 7 after injection, the eyes were enucleated and processed for the biochemical, and histological analyses as described below.

### 4.3. Histology, Imaging, and Retinal Degeneration Quantification

Enucleated eyes were fixed in 2% paraformaldehyde, 2.5% glutaraldehyde, and 5% CaCl_2_ made in 0.1 M cacodylate buffer overnight at 4 °C. The anterior segments were removed under a dissecting microscope, and the posterior eyecups were processed for epon embedding as previously described [17]. Briefly, fixed eyecups were post-fixed in 1% osmium tetroxide made in 0.1 M cacodylate buffer, dehydrated in graded series from ethyl alcohol, embedded in epon resin and polymerized for 48 h at 60 °C. For bright field imaging, semi-thin sections were cut using a diamond histotech knife (DiATOME, Leica Biosystems, Chicago, IL, USA), collected on glass slides, and stained with toluidine blue. Sections were imaged either with a Zeiss Axioimager Z1 with MRc5 camera (Carl Zeiss AG, Oberkochen, Germany) or a Leica Thunder K3 camera (Leica Biosystems, Exton, PA, USA). Images were acquired ~200 µm from the optic nerve on both sides. Images were exported to ImageJ 1.43 software (NIH, Bethesda, MD, USA) and were calibrated from a reference scale embedded in the images. Eyes were cut until the optic nerve was visible. Degeneration was quantified from images of the entire posterior pole (half eyecup within the optic nerve head). Next, the total (area of retina on both sides of the optic nerve) and degenerated (areas with absent and abnormal RPE) retinal areas were delineated using the freehand line tool. Finally, degenerated areas were calculated for each sample according to the following formula: degenerated area = (degenerated area/total whole-mounts area) × 100, as previously described [27].

### 4.4. Protein Extraction and Western Blotting

Under a dissecting microscope, the retina was mechanically detached from the RPE. RPE isolation followed a previously described method that demonstrated that RPE lysates contained abundant RPE65 protein, an RPE-specific marker, as assessed by Western blot analysis [80]. Mechanically isolated retinas and RPE were lysed in RIPA buffer (Alfa Aesar, Havermill, MA, USA) containing proteinase (P8340, Sigma-Aldrich, St. Louis, MO, USA) and phosphate inhibitors (P5726, P0044, Sigma-Aldrich, St. Louis, MO, USA). Retinas were sonicated twice for 15 s and RPE was lysed using 27 1/2 G syringe needle. After lysing with a syringe needle, RPE lysate was incubated on ice and vortexed every 5 min. After lysis, retina and RPE lysates were centrifuged at 4 °C for 10 min at 14,000 rpm, the supernatants were transferred to a clean Eppendorf tube and processed for protein quantification using MicroBCA Kit (Thermo Scientific, Waltham, MA, USA). An amount of 30 µg of protein for retina and RPE lysate were separated on 4–20% Tris-Glycine SDS PAGE (Bio-rad, San Francisco, CA, USA) and transferred on PVDF membrane (Immobilon-FL, Merck Millipore Ltd., Burlington, MA, USA) under wet conditions at a constant 100 volts for an hour. Immunoblotting membranes were reacted with the following antibodies: anti-ERK1/2 (rabbit polyclonal, 4695S), anti-phospho ERk1/2 (rabbit polyclonal, 9101S), anti-P38 (rabbit polyclonal, 4511S), anti-pP38 (rabbit polyclonal, 9212S), anti-JNK (rabbit polyclonal, 9252S), anti-pJNK (rabbit polyclonal, 9251S), anti-FOXO1 (rabbit monoclonal, C29H4), and β-actin (mouse monoclonal 8H10D10); all antibodies were purchased from Cell Signaling Technology, Danvers, MA, USA. The following secondary antibodies provided by Licor Biosciences (Lincoln, NE, USA) were used: anti-rabbit IRDye^®^680RD and anti-rabbit IRDye^®^800CW. The immunoreactive bands were visualized using Oddessey CLx (Licor Biosciences, Lincoln, NE, USA). The bands were quantified in ImageJ 1.43 software. The density of pERK1/2, pP38 and pJNK1/2 was normalized to ERK, P38 and JNK1/2 of the same sample, and C57BL/6J mice injected with PBS were used to normalize the calculated fold changes.

### 4.5. Immunohistochemistry

Enucleated eyes were directly placed in Tissue-Tek O.C.T Compound (4583; Sakura Finetek, Tokyo, Japan) and flash frozen in liquid nitrogen. Cryosections (8 µm) were cut on a cryostat HM 505E (Microm, Walldorf, Germany) equipped with a CryoJane tape transfer system (Leica Microsystems Inc., Chicago, IL, USA). For labeling, sections were washed, blocked in PBS supplemented with 1% BSA (PBS/BSA) for 30 min, and incubated with primary antibodies overnight. The next day, sections were incubated in secondary antibodies coupled to Alexa 488 or 594 (1:1000 dilution). Nuclei were labeled with TO-PRO-3 (Molecular Probes Inc., Life Technologies, Eugene, OR). Sections were analyzed using the Leica TCS-SP8 (Wetzlar, Germany). The antibodies used included anti-active caspase 3 (1:150; rabbit, 559565, BD Pharmingen, San Diego, CA, USA), anti-pMLKL (1:250; rabbit, ab196436, Abcam, Cambridge, Boston, MA, USA), and anti-Daxx (H-7) Alexa flour 488 (1:50; mouse, SC-8043, Santa Cruz biotechnology, Dallas, TX, USA). Micrographs were acquired using the same acquisition parameters and calibrated from a scale reference embedded in the image.

### 4.6. Programmed Cell Death Treatments

A cohort of C57BL and DJ-1 KO mice (3 months) were first intravenously injected with 10 and 20 mg/kg NaIO_3_ and returned to vivarium. After one hour, these mice were either injected with 5% DMSO prepared in D-PBS (vehicle) or 2 mg/kg ZVAD-fmk prepared in D-PBS (ALX-260-020, Enzo Life Sciences Inc., Farmingdale, NY, USA) intraperitoneally (IP) consecutively for 7 days (Day 0–Day 6) as previously reported [81]. On day 7, the mice were euthanized, and eyes were enucleated and processed for further analysis.

For Necrostatin-1 (Nec-1) treatment, mice were first intravenously injected with 10 and 20 mg/kg NaIO_3_ and, after 1 h, were intravenously (IV) injected with either 5% DMSO prepared in D-PBS (vehicle) or 3.5 mg/kg Nec-1 prepared in D-PBS (N9037, Sigma-Aldrich, St. Louis, MO, USA) as previously reported [82]. On Day 7, mice were euthanized, and eyes were enucleated and processed for further analysis.

### 4.7. Statistical Analysis

All data were analyzed for statistical significance using GraphPad Prism v9.0 (GraphPad Software, La Jolla, CA, USA). Student’s *t*-tests were used for determining statistical significance between groups with an alpha value of 0.05. *p* values ≤ 0.05 were considered statistically significant.

## Data Availability

The data presented in this study are available in the article and on request from the corresponding author.

## Supplementary Materials

The following supporting information can be downloaded at https://www.mdpi.com/article/10.3390/ijms27062541/s1.

## Abbreviations

The following abbreviations are used in this manuscript:

AMD	Age-related macular degeneration
RPE	Retinal pigment epithelium
NaIO_3_	Sodium iodate
MAPK	Mitogen-activated protein kinase
DJ-1 KO	DJ-1 knockout
MAPKs	Mitogen-activated protein kinases
ERK	Extracellular signal-regulated kinase
JNK	c-Jun N-terminal kinases
AGEs	Advanced glycation end products
FOXO1	Forkhead box protein O1
Daxx	Death domain-associated protein
ASK1	Apoptosis signal-regulating kinase 1
IS	Photoreceptor inner segments
GCL	Ganglion cell layer
IPL	Inner plexiform layer
OPL	Outer plexiform layer
INL	Inner nuclei layer
acasp3	Active caspase 3
MLKL	Mixed lineage kinase domain-like pseudo kinase
Nec-1	Necrostatin-1
ROS	Reactive oxygen species
PI3K	Phosphatidylinositol-3-kinase
