# Supplementary material for: Mechanisms of Programmed Cell Death in Sodium Iodate-Driven Retinal Degeneration and the Role of DJ-1
Source: Int J Mol Sci. 2026 Mar 10;27(6):2541. doi: 10.3390/ijms27062541 (PMC13026112; doi:10.3390/ijms27062541)
Supplement: Supplementary file 1 [file ijms-27-02541-s001.zip › ijms-4112052-supplementary.pdf]

Figure S1. Representative images of immunoreactivity of Daxx (green) in the retinas of C57BL **(A-C)** and DJ-1 KO **(D-F)** mice injected with $NaIO_3$; red: TO-PRO-3; bar = 40 µm.

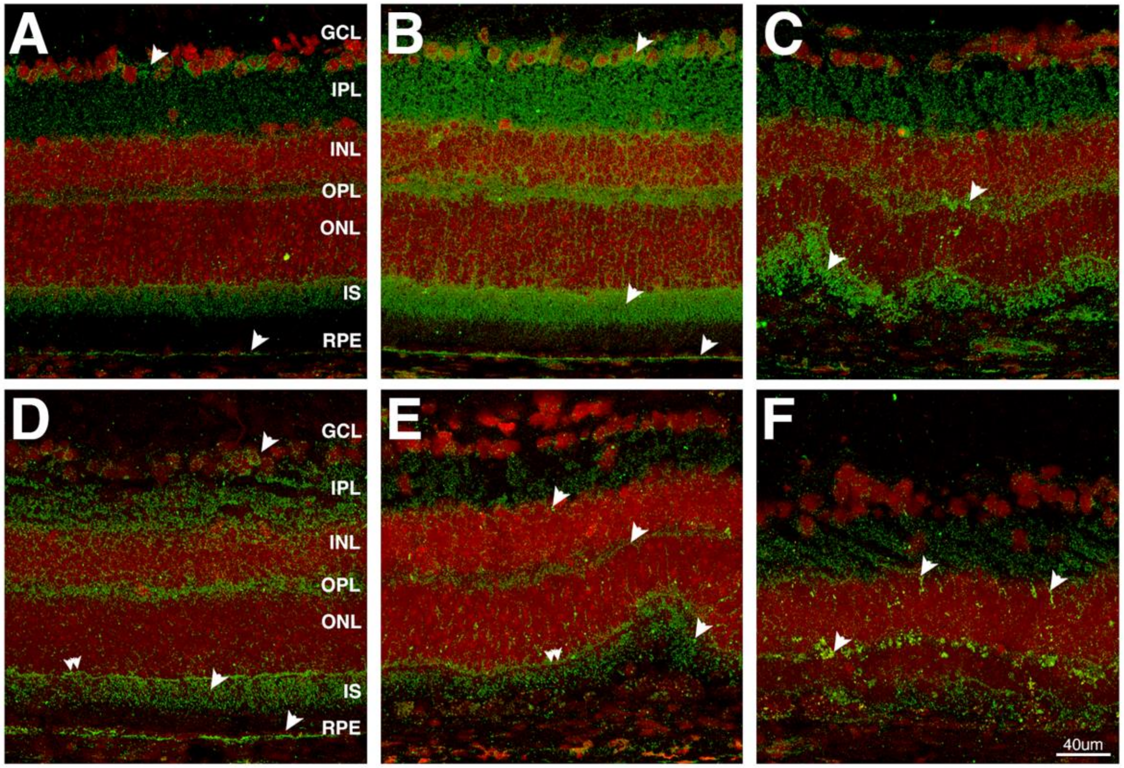

Table S1. Detailed list of the 224 proteins altered in the RPE of the DJ-1 KO mice with the diseases and functions annotated to them.

Categories	Diseases of Function Annotation	p-value	Molecules	No Molecules
Cancer,Cell Death and Survival,Organismal Injury and Abnormalities,Tumor Morphology	Cell death of osteosarcoma cells	3.64E-21	CHMP4B,COPB2,EEF1A1,EIF3L,NUP155,NUP214,PSMA5,PSMA6,PSMA7,PUF60,RAN,RPL11,RPL13,RPL13A,Rpl23a,RPL3, Rpl32,RPL35,RPL6,RPL7,RPL7A,RPS14,RPS15A,RPS17,RPS24,RPS3,RPS7,SF3B3,U2AF2,VDAC2	30
Cell Death and Survival	Necrosis	1.94E-16	AARS1,ABCC9,ACP1,ALDH1A1,ALDH1L1,ANPEP,APOE,ARG1,ARMC10,ATG7,BGN,CALR,CAMK2D,CASP8,CBX3,CCT3,CD200,CD47,CD82,CHMP4B,CHMP6,COL1A1,COL6A1,COMT,COPB2,COPZ1,COX7A2L,CTSD,DAB2,DCN,DCT,DCTN2,DDX3X,DHX9,DIABLO,DNAJA3,DSP,DYNC1H1,EEF1A1,EGFR,EIF1AX,EIF3L,EIF6,ELAVL1,ELOC,ENO1,ETHE1,F2,F3,FABP3,FADD,FDPS,FKBP4,FN1,FUBP1,Fus,G6PC3,GCLC,GFPT1,GGT1,GLUD1,GNAI2,GNB2,GSTM5,GSTZ1,HGS,HK2,HNRNPH1,HNRNPK,HSP90B1,HSPA5,HSPD1,ITGA6,JUP,LGALS7/LGALS7B,LMNB1,LUM,M6PR,MAPK1,MCU,MET,MGP,MKI67,MMP2,MSI2,MTCH2,MYBBP1A,MYH10,MYH4,MYO6,MYOC,NAE1,NDUFV2,NEK7,NUP155,NUP214,OPA1,PAFAH1B1,PAK2,PARK7,PDK2,PGRMC1,PPM1A,PPP2R1A,PPP3R1,PPP5C,PRKACA,PRPH,PSAP,PSMA5,PSMA6,PSMA7,PTGES,PTRH2,PUF60,Pzp2,RALB,RAN,RBBP4,RDX,RECK,RHOA,ROCK1,ROCK2,RPL11,RPL13,RPL13A,Rpl23a,RPL3,Rpl32,RPL35,RPL6,RPL7,RPL7A,RPS14,RPS15A,RPS17,RPS24,RPS3,RPS6KA3,RPS7,RPSA,S100A10,S100A6,SAR1A,SBDS,SDHB,SF3B3,SIRT2,SLC1A1,SLC25A12,SLC3A2,SLK,SNCA,SNX1,SORT1,SPNS1,STRA6,STUB1,TAF4B,TARDBP,TGM2,THY1,TJP2,TMED10,TRIM25,TTR,TUFM,TXN2,U2AF2,UBA1,UPF1,USP47,VASN,VDAC1,VDAC2,VTI1B,XRCC6	178
Cancer,Cell Death and Survival,Orga	Necrosis of malignant tumor	1.54E-11	ANPEP,ATG7,CASP8,CHMP4B,COPB2,DIABLO,EEF1A1,EGFR,EIF3L,ENO1,FADD,MET,NUP155,NUP214,PSMA5,PSMA6,PSMA7,PUF60,RALB,RAN,RPL11,RPL13,RPL13A,Rpl23a,RPL3	39

nismal Injury and Abnormalities ,Tumor Morphology			,Rpl32,RPL35,RPL6,RPL7,RPL7A,RPS14,RPS15A,RPS17,RPS24,RPS3,RPS7,SF3B3,U2AF2,VDAC2	
Cancer,Cell Death and Survival,Organismal Injury and Abnormalities ,Tumor Morphology	Cell death of cancer cells	3.72E-11	ATG7,CASP8,CHMP4B,COPB2,DIABLO,EEF1A1,EGFR,EIF3L,ENO1,FADD,MET,NUP155,NUP214,PSMA5,PSMA6,PSMA7,PUF60,RALB,RAN,RPL11,RPL13,RPL13A,Rpl23a,RPL3,Rpl32,RPL35,RPL6,RPL7,RPL7A,RPS14,RPS15A,RPS17,RPS24,RPS3,RPS7,SF3B3,U2AF2,VDAC2	38
Cancer,Cell Death and Survival,Organismal Injury and Abnormalities ,Tumor Morphology	Cell death of tumor cells	1.21E-10	ANPEP,ATG7,CASP8,CD47,CHMP4B,COPB2,DIABLO,EEF1A1,EGFR,EIF3L,ENO1,F2,FADD,MAPK1,MET,NUP155,NUP214,PSMA5,PSMA6,PSMA7,PUF60,RALB,RAN,RECK,RHOA,RPL11,RPL13,RPL13A,Rpl23a,RPL3,Rpl32,RPL35,RPL6,RPL7,RPL7A,RPS14,RPS15A,RPS17,RPS24,RPS3,RPS7,SF3B3,U2AF2,VDAC2	44
Cell Death and Survival	Apoptosis	8.06E-09	AAMDC,AARS1,ACIN1,AKAP8,ALDH1A1,ALDH1L1,ANPEP,ANXA11,APCS,APOE,ARG1,ARHGEF12,ARMC10,ATG7,ATOX1,BGN,BLVRA,BTF3,C1QBP,CALR,CAMK2D,CASP8,CBX3,CD200,CD47,CD82,COL1A1,COPZ1,COX7A2L,CTSD,DAB2,DAD1,DCN,DCT,DDB1,DDX3X,DHX9,DIABLO,DNAJA3,DSP,DYNC1H1,EEF1A1,EGFR,EIF4G1,EIF6,ELAVL1,ELOC,ENO1,ETHE1,F2,F3,FADD,FKBP4,FN1,FUBP1,Fus,G6PC3,GCLC,GFPT1,GGT1,GLUD1,GNAI2,GNB2,GNG5,GSTM5,GSTZ1,HIGD1A,HK2,HNRNPH1,HNRNPK,HSP90B1,HSPA5,HSPD1,ITGA6,JUP,LGALS7/LGALS7B,LMNB1,LRP2,LUM,MAPK1,MET,MGP,MKI67,MMP2,MPRIP,MTCH2,MYBBP1A,MYH10,MYO6,MYOC,NAE1,NCKAP1,NDUFV2,NEK7,OPA1,PAFAH1B1,PAK2,PARK7,PDCD10,PLCD1,PPM1A,PPM1F,PPP2R1A,PPP3R1,PPP5C,PRDX4,PRKACA,PRPH,PSAP,PTGES,PTRH2,PUF60,RALB,RBBP4,RDX,RHOA,ROCK1,ROCK2,RPS24,RPS3,RPS6KA3,S100A10,S100A6,SAR1A,SBDS,SERPINB1,SIRT2,SLK,SMARCA5,SNCA,SNX1,SORT1,STRA6,STUB1,TAF4B,TARDBP,TGM2,THY1,TJP2,TM7SF3,TMED10,TRIM25,TTR,TXN2,UBA1,UPF1,USP47,USP7,VASN,VDAC1,VDAC2,VTI1B,XRCC6	153

Cell Death and Survival,Organismal Injury and Abnormalities,Renal and Urological Disease	Necrosis of kidney	2,16E-08	APOE,ATG7,BGN,CASP8,CHMP6,DCN,DCTN2,EEF1A1,EGFR,F2,F3,FADD,GFPT1,GNB2,GSTM5,GSTZ1,HK2,HSPA5,MYOC,PAK2,PARK7,PPM1A,PPP3R1,PPP5C,RALB,RHOA,SIRT2,SLK,SNCA,SPNS1,STRA6,STUB1,TARDBP,TJP2,VDAC1	35
Cell Death and Survival,Organismal Injury and Abnormalities,Renal and Urological Disease	Cell death of kidney cells	2.2E-08	APOE,ATG7,BGN,CASP8,CHMP6,DCN,DCTN2,EEF1A1,EGFR,F2,F3,FADD,GFPT1,GNB2,GSTM5,HK2,HSPA5,MYOC,PAK2,PARK7,PPM1A,PPP3R1,PPP5C,RALB,RHOA,SIRT2,SLK,SNCA,SPNS1,STRA6,STUB1,TARDBP,TJP2,VDAC1	34
Cell Death and Survival	Cell viability of tumor cell lines	4.2E-08	AK3,ANAPC1,APOE,ARFGEF2,ATG7,CA2,CALR,CAMK2D,CASP8,CD200,CD82,COL1A1,COPB2,CTSD,DCT,DHX9,DIABLO,DPP3,EGFR,EIF4A1,EIF4A3,EIF4G1,ELAVL1,ENO1,FADD,FAM120A,FKBP4,FN1,FUBP1,GCLC,GLUD1,HK2,HSP90B1,HSPA5,ITGA6,LGALS7/LGALS7B,LMNB1,LSM8,MAPK1,MET,NEK7,NUP155,PARK7,PFDN2,PGRMC1,PPP2R1A,PRKACA,PRPH,PSAP,PSMA1,PSMA5,PSMA6,PTPN23,RBBP4,RHOA,RPS6KA3,RPSA,S100A6,SDCBP,SF3B3,SNCA,SRSF3,TARDBP,TGM2,U2AF2,UPF1,USP47,XRCC6	68
Cell Death and Survival,Organismal Injury and Abnormalities,Renal and Urological Disease	Cell death of kidney cell lines	1.76E-07	ATG7,CASP8,CHMP6,DCTN2,EEF1A1,EGFR,F2,F3,FADD,GNB2,GSTM5,HK2,HSPA5,MYOC,PAK2,PARK7,PPM1A,PPP3R1,RALB,RHOA,SIRT2,SLK,SNCA,SPNS1,STRA6,STUB1,TARDBP,TJP2,VDAC1	29

Cell Death and Survival	Anoikis	1.88E-06	CALR,CASP8,CD82,DAB2,DIABLO,EEF1A1,EGFR,FADD,FN1,MAPK1,PTRH2,RDX,RHOA,SNX1,TGM2	15
Cell Death and Survival,Organismal Injury and Abnormalities	Cell death of epithelial cells	7.79E-06	ALDH1A1,ATG7,CALR,CASP8,DAB2,DCN,DCTN2,EEF1A1,EGFR,F2,F3,FADD,FKBP4,HK2,HSPA5,HSPD1,ITGA6,LGALS7/LGALS7B,MAPK1,MET,MMP2,MTCH2,MYOC,PAK2,PARK7,PPM1A,PPP3R1,PPP5C,RHOA,SIRT2,SNCA,SPNS1,TARDBP,TGM2,TJP2,VASN,VDAC1	37
Cell Death and Survival,Organismal Injury and Abnormalities	Cell death of epithelial cell lines	1.31E-05	ATG7,CASP8,DAB2,DCTN2,EEF1A1,EGFR,F2,FADD,HK2,HSPA5,HSPD1,MAPK1,MET,MYOC,PAK2,PARK7,PPM1A,PPP3R1,RHOA,SIRT2,SNCA,SPNS1,TARDBP,TJP2,VDAC1	25
Cell Death and Survival	Cell viability	1.43E-05	AK3,ANAPC1,APOE,ARFGEF2,ATG7,CA2,CALR,CAMK2D,CASP8,CD200,CD47,CD82,CDH13,COL1A1,COPB2,CTSD,DAB2,DCT,DHX9,DIABLO,DPP3,EGFR,EIF4A1,EIF4A3,EIF4G1,ELAVL1,ENO1,F2,FADD,FAM120A,FKBP4,FN1,FUBP1,GCLC,GLUD1,HK2,HNRNPK,HSP90B1,HSPA5,HSPD1,IQGAP1,ITGA6,LGALS7/LGALS7B,LMNB1,LSM8,MAPK1,MET,NEK7,NUP155,PAK2,PARK7,PFDN2,PGRMC1,PNPLA6,POLDIP2,PPM1A,PPP2R1A,PRKACA,PRPH,PSAP,PSMA1,PSMA5,PSMA6,PTPN23,RALB,RBBP4,RHOA,RPS6KA3,RPSA,S100A6,SDCBP,SF3B3,SMC3,SNCA,SRSF3,TAF4B,TARDBP,TGM2,U2AF2,UPF1,USP47,VDAC1,XRCC6	83
Cell Death and Survival,Embryonic Development	Cell death of embryonic cell lines	1.66E-05	ATG7,CASP8,DCTN2,DIABLO,DNAJA3,EEF1A1,EGFR,F2,FADD,FKBP4,FN1,HK2,HSPA5,MTCH2,MYOC,OPA1,PAK2,PARK7,PPM1A,PPP3R1,PTRH2,RHOA,SIRT2,SNCA,SPNS1,STUB1,TARDBP,TJP2,VDAC1	29
Cell Death and Survival	Cell survival	1.94E-05	AK3,ANAPC1,APOE,ARFGEF2,ATG7,CA2,CALR,CAMK2D,CASP8,CD200,CD47,CD82,CDH13,COL1A1,COPB2,CTSD,DAB2,DCT,DDX3X,DHX9,DIABLO,DPP3,EGFR,EIF4A1,EIF4A3,EIF4G1,ELAVL1,ENO1,F2,FADD,FAM120A,FKBP4,FN1,FUBP1,GCLC,GLUD1,HK2,HNRNPK,HSP90B1,HSPA5,HSPD1,IQGAP1,ITGA6,JUP,LGALS7/LGALS7B,LMNB1,LSM8,MAPK1,MET,NEK7,NUP155,PAK2,PARK7,PFDN2,PGRMC1,PNPLA6,POLDIP2,PPM1A,PPP2R1A,PRKACA,PRPH,PSAP,PSMA1,PSMA5,PSMA6,PSMA7,PTPN23,RALB,RBBP4,RHOA,RPS6KA3,RPSA,S100A6,SDCBP,SF3B3,SMC3,SNCA,SRSF3,TAF4B,TARDBP,TGM2,U2AF2,UPF1,USP47,VDAC1,XRCC6	86

Cell Death and Survival	Cell viability of neuroblastoma cell lines	0.000023	APOE,DIABLO,DPP3,HK2,PRPH,PSAP,S100A6,SNCA,TARDBP,TGM2	10
Cell Death and Survival	Cell death of central nervous system cells	2.42E-05	APOE,ATG7,CASP8,CTSD,F2,FADD,GCLC,HK2,HSPA5,HSPD1,MAPK1,NAE1,PARK7,PRPH,PTGES,RHOA,ROCK1,ROCK2,RPS3,SLC1A1,SLC25A12,SNCA,TARDBP,TGM2	24
Cell Death and Survival	Cell death of fibroblast cell lines	3.26E-05	ATG7,CASP8,CTSD,DDX3X,DIABLO,DNAJA3,EEF1A1,EGFR,EIF6,ENO1,FADD,FKBP4,FN1,GNB2,GSTM5,HSPA5,HSPD1,ITGA6,MAPK1,MET,MTCH2,OPA1,PAK2,PARK7,PTRH2,RALB,RHOA,STUB1,TGM2,UBA1,VDAC1	31
Cell Death and Survival	Apoptosis of cervical cancer cell lines	3.3E-05	CASP8,DIABLO,DNAJA3,DYNC1H1,EEF1A1,EGFR,ELAVL1,ELOC,FADD,HNRNPH1,HNRNPK,LGALS7/LGALS7B,MAPK1,NEK7,OPA1,PAK2,PTRH2,PUF60,RALB,RPS24,RPS6KA3,SNCA,VDAC1,XRCC6	24
Cell Death and Survival	Cell death of cervical cancer cell lines	4.47E-05	ATG7,CALR,CASP8,DHX9,DIABLO,DNAJA3,DYNC1H1,EEF1A1,EGFR,ELAVL1,ELOC,FADD,HNRNPH1,HNRNPK,LGALS7/LGALS7B,MAPK1,NEK7,OPA1,PAK2,PPP2R1A,PTRH2,PUF60,RALB,RPS24,RPS6KA3,SNCA,VDAC1,XRCC6	28
Cell Death and Survival,Organismal Injury and Abnormalities	Necrosis of epithelial tissue	4.55E-05	ALDH1A1,ATG7,CALR,CASP8,DAB2,DCN,DCTN2,EEF1A1,EGFR,F2,F3,FADD,FKBP4,HK2,HSPA5,HSPD1,ITGA6,LGALS7/LGALS7B,LUM,MAPK1,MET,MMP2,MTCH2,MYOC,PAK2,PARK7,PPM1A,PPP3R1,PPP5C,PRKACA,RDX,RHOA,SIRT2,SNCA,SPNS1,TARDBP,TGM2,TJP2,VASN,VDAC1	40
Cell Death and Survival,Skeletal and Muscular Disorders	Necrosis of muscle	5.21E-05	ABCC9,CALR,CAMK2D,CASP8,COL1A1,COL6A1,DSP,EEF1A1,ELAVL1,FADD,GNAI2,HK2,HSPD1,MAPK1,MKI67,MMP2,MYH10,MYH4,NDUFV2,PARK7,RHOA,RPSA,S100A6,SLK,SORT1,STUB1	26
Cell Death and Survival	Anoikis of tumor cell lines	5.46E-05	CALR,CASP8,CD82,DAB2,FN1,MAPK1,PTRH2,RHOA,SNX1	9
Cell Death and	Cell death of brain cells	5.72E-05	APOE,ATG7,CASP8,CTSD,F2,FADD,GCLC,HK2,HSPA5,HSPD1,NAE1,PARK7,PTGES,RHOA,ROCK1,ROCK2,RPS3,SLC1A1,SLC25A12,SNCA,TARDBP,TGM2	22

Survival,Neurological Disease,Organismal Injury and Abnormalities				
Cell Death and Survival	Cell death of brain	5.86E-05	POE,ATG7,CASP8,CTSD,F2,FADD,GCLC,HK2,HSPA5,HSPD1,NAE1,PARK7,PTGES,RHOA,ROCK1,ROCK2,RPS3,SDHB,SLC1A1,SLC25A12,SNCA,TARDBP,TGM2	23
Cell Death and Survival,Skeletal and Muscular Disorders	Cell death of muscle cells	8.95E-05	ABCC9,CALR,CAMK2D,CASP8,COL1A1,DSP,EEF1A1,ELAVL1,FADD,GNAI2,HK2,HSPD1,MAPK1,MKI67,MMP2,MYH10,MYH4,NDUFV2,PARK7,RHOA,RPSA,S100A6,SLK,SORT1,STUB1	25
Cell Death and Survival	Apoptosis of tumor cell lines	0.000092	ALDH1L1,APOE,ARG1,ARMC10,ATG7,CALR,CASP8,CBX3,CD47,CD82,COL1A1,COPZ1,COX7A2L,CTSD,DAB2,DCT,DHX9,DIABLO,DNAJA3,DSP,DYNC1H1,EEF1A1,EGFR,ELAVL1,ELOC,ENO1,ETHE1,FADD,FN1,FUBP1,GGT1,HK2,HNRNPH1,HNRNPK,HSPA5,HSPD1,JUP,LGALS7/LGALS7B,LMNB1,MAPK1,MET,MYBBP1A,MYO6,NEK7,OPA1,PAK2,PARK7,PPP2R1A,PPP5C,PRKACA,PSAP,PTRH2,PUF60,RALB,RHOA,RPS24,RPS3,RPS6KA3,S100A6,SAR1A,SBDS,SNCA,SNX1,SORT1,STUB1,TGM2,THY1,TMED10,TRIM25,TXN2,VDAC1,VDAC2,XRCC6	73
Cell Death and Survival	Cell viability of myeloma cell lines	0.000101	AK3,CD82,COPB2,EIF4A1,EIF4A3,EIF4G1,HSP90B1,MET,PSMA1,PSMA6,RPS6KA3	11
Cell Death and Survival,Neurological Disease,Organismal Injury and Abnormalities	Cell death of cerebral cortex cells	0.000105	APOE,CASP8,CTSD,F2,FADD,GCLC,HK2,HSPA5,HSPD1,NAE1,PARK7,PTGES,RHOA,RPS3,SLC1A1,SLC25A12,SNCA,TARDBP,TGM2	19
Cell Death and Survival	Cell death of squamous cell	0.000115	CALR,CASP8,EGFR,FADD,FN1,HNRNPH1,JUP,MAPK1,MET,THY1	10

	carcinoma cell lines			
Cell Death and Survival	Cell death of tumor cell lines	0.000159	ALDH1L1,APOE,ARG1,ARMC10,ATG7,CALR,CASP8,CBX3,CCT3,CD47,CD82,COL1A1,COPZ1,COX7A2L,CTSD,DAB2,DCT,DDX3X,DHX9,DIABLO,DNAJA3,DSP,DYNC1H1,EEF1A1,EGFR,EIF1AX,ELAVL1,ELOC,ENO1,ETHE1,FADD,FN1,FUBP1,GGT1,HK2,HNRNPH1,HNRNPK,HSPA5,HSPD1,ITGA6,JUP,LGALS7/LGALS7B,LMNB1,MAPK1,MCU,MET,MSI2,MYBBP1A,MYO6,NEK7,OPA1,PAK2,PARK7,PDK2,PGRMC1,PPP2R1A,PPP5C,PRKACA,PSAP,PTRH2,PUF60,RALB,RDX,RHOA,RPS24,RPS3,RPS6KA3,S100A6,SAR1A,SBDS,SF3B3,SLC3A2,SNCA,SNX1,SORT1,STUB1,TARDBP,TGM2,THY1,TMED10,TRIM25,TUFM,TXN2,USP47,VDAC1,VDAC2,XRCC6	87
Cell Death and Survival	Cell death of connective tissue cells	0.000167	ATG7,BGN,CASP8,CTSD,DCN,DDX3X,DIABLO,DNAJA3,EEF1A1,EGFR,EIF6,ENO1,FADD,FKBP4,FN1,GNB2,GSTM5,HSPA5,HSPD1,ITGA6,LUM,M6PR,MAPK1,MET,MGP,MTCH2,OPA1,PAK2,PARK7,PTRH2,RALB,RHOA,SLK,STUB1,TGM2,UBA1,VDAC1,XRCC6	38
Cell Death and Survival	Cell death of neuroblastoma cell lines	0.000221	APOE,ATG7,CASP8,CCT3,DHX9,DIABLO,EGFR,ENO1,FADD,HSPA5,PARK7,S100A6,SNCA,STUB1,TARDBP,TGM2	16
Cell Death and Survival,Organismal Injury and Abnormalities	Anoikis of endothelial cells	0.000239	CASP8,FADD,RDX	3
Cell Death and Survival,Organismal Injury and Abnormalities	Apoptosis of epithelial cell lines	0.000295	CASP8,DAB2,EEF1A1,EGFR,F2,FADD,HK2,HSPA5,HSPD1,MAPK1,MET,MYOC,PAK2,PPM1A,PPP3R1,SNCA,TJP2,VDAC1	18
Cardiovascular Disease,Cell Death and Survival,Organismal Injury and	Cell death of cardiomyocytes	0.000296	ABCC9,CALR,CAMK2D,CASP8,DSP,ELAVL1,FADD,GNAI2,HK2,HSPD1,MAPK1,MMP2,MYH10,PARK7,RHOA,RPSA,S100A6	17

Abnormalities ,Skeletal and Muscular Disorders				
Cell Death and Survival,Cell Morphology, Nervous System Development and Function,Neurological Disease	Loss of neuroglia	0.000325	AFDN,HSPA5,LRP2,SNCA	4
Cell Death and Survival,Organismal Injury and Abnormalities ,Renal and Urological Disease	Apoptosis of kidney cell lines	0.000345	CASP8,EEF1A1,F2,F3,FADD,GNB2,HSPA5,MYOC,PAK2,PPM1A,PPP3R1,RALB,SLK,SNCA,STRA6,STUB1,TJP2,VDAC1	18
Cell Death and Survival	Apoptosis of neurons	0.000401	AARS1,APOE,CASP8,CD200,DIABLO,F2,FADD,FN1,Fus,GCLC,GLUD1,GSTM5,HK2,HSPA5,HSPD1,MAPK1,MET,NAE1,PAFAH1B1,PARK7,PRKACA,PRPH,RHOA,ROCK1,RPS3,SNCA,TTR,VTI1B,XRCC6	29
Cell Death and Survival	Neuronal cell death	0.000469	AARS1,APOE,ATG7,CASP8,CD200,CTSD,DIABLO,EGFR,F2,FADD,FN1,Fus,GCLC,GLUD1,GSTM5,HGS,HK2,HSPA5,HSPD1,MAPK1,MET,NAE1,PAFAH1B1,PARK7,PRKACA,PRPH,RHOA,ROCK1,ROCK2,RPS3,SDHB,SLC1A1,SLC25A12,SNCA,SORT1,TARDBP,TGM2,TTR,UPF1,VTI1B,XRCC6	41
Cell Death and Survival,Orga	Anoikis of dermal cells	0.000542	EGFR,TGM2	2

nismal Injury and Abnormalities				

Table S2. Detailed list of the 151 proteins altered in the retina of the DJ-1 KO mice with the diseases and functions annotated to them.

Categories	Diseases of Function Annotation	p-value	Molecules	No Molecules
Cell Death and Survival	Necrosis	1.08E-10	AAK1,AARS1,ACSL1,AKAP12,ALB,ALDH1A1,ANXA1,ANXA5,AP2M1,APOA1,APOE,ARHGDIA,ATP1A2,ATP2A2,ATP2B2,ATP2C1,BBS4,CAMK2B,CAPN2,CAV1,CBR1,CCAR1,CIAPIN1,CLTC,COX7A2L,CRYAA/CRYAA2,CTBP2,DDX3X,DGKE,DHODH,DHX9,DIDO1,DMD,DPYSL3,DYNC1H1,EHD4,EIF5B,EXOG,EZR,FLNA,GAD2,GPR37,GSN,GSTM5,HADHA,HCFC1,HDAC6,HK2,HMOX2,HSP90AA1,HSPA1A/HSPA1B,HSPA5,HSPB1,IDE,KIF1A,KIF3A,LDHA,MAP2K2,MAP4,MARCHF5,MEF2D,MVP,NDUFA13,NFYA,NGFR,NPM1,NQO2,NUDCD3,NUP155,NUP54,NXF1,OGFOD1,PAFAH1B1,PARK7,PDCD5,PDCD6IP,PHGDH,PKM,PLRG1,POR,PRPF8,PSMD6,PSME3,PTPN11,RABGEF1,RPL27,RPL27A,RPL3,RPS12,RPS13,RPS16,RPS6KA3,RTN4,SARNP,SERPINA1,Sf3a2,SF3B1,SLC25A12,SLC3A2,SMAD2,SMPD2,Snrpg,SRCIN1,STAM,SUPT6H,TAGLN2,TCP1,TF,TMEM109,TPD52,TPT1,TXN2,USP47,VCAM1,WDR5,WFS1,WTAP,XPO1	118
Cell Death and Survival	Apoptosis	1.77E-08	AARS1,ACSL1,AGO1,AGO2,AKAP12,ALB,ALDH1A1,ANXA1,ANXA5,ANXA6,AP2M1,APOA1,APOE,ARHGDIA,ATP1A2,ATP2B2,ATP2C1,BBS2,BBS4,CAPN2,CAV1,CCAR1,CIAPIN1,CIRBP,CKMT1A/CKMT1B,CLTC,COX7A2L,CRYAA/CRYAA2,CTBP2,CUL5,DDRGK1,DDX19A,DDX3X,DGKE,DHODH,DHX9,DIDO1,DYNC1H1,EHD1,EHD4,ERC1,EXOG,EZR,FLNA,Folh1,GPM6B,GPR37,GSN,GSTM5,HCFC1,HDAC6,HIGD1A,HK2,HMOX2,HSP90AA1,HSPA1A/HSPA1B,HSPA5,HSPB1,IDE,KDM1A,KIF3A,LDHA,MAP2K2,MAP4,MARCHF5,MEF2D,MVP,MYO18A,NDUFA13,NFYA,NGFR,NPM1,NQO2,OGFOD1,PAFAH1B1,PARK7,PDCD5,PDCD6IP,PHGDH,PKM,PLRG1,POR,PSMD6,PTPN11,RANBP1,RBP3,RPS6KA3,RTN4,SAFB,SELENOF,SERPINA1,SF3B1,SIN3A,SLC2A3,SMAD2,SMPD2,SRCIN1,TAGLN2,TCP1,TDRD7,TF,TMEM109,TOMM40,TPD52,TPT1,TXN2,USP47,WDR5,WFS1,WTAP,XPO1	111
Cancer,Cell Death and Survival,Organismal Injury and	Cell death of osteosarcoma cells	2.16E-07	NUP155,NUP54,NXF1,PRPF8,RPL27,RPL27A,RPL3,RPS12,RPS13,RPS16,Sf3a2,SF3B1,Snrpg	13

Abnormalities ,Tumor Morphology				
Cell Death and Survival	Cell survival	4.36E-05	AGO2,ALB,ANXA5,APOE,ATP1A2,ATP2C1,CAMK2B,CAPN2,CAV1,CBR1,CCAR1,CIAPIN1,DDX3X,DHX9,DNAJB6,EHD4,EZR,FLNA,GAD2,GPR37,HK2,HMGN1,HMOX2,HSPA1A/HSPA1B,HSPA5,HSPB1,IDE,IK,KDM1A,KIF1A,LDHA,MVP,MYO18A,NDUFA13,NGFR,NUP155,OPN1SW,PARK7,PKM,PLRG1,POR,PPFIA2,PPIB,PRPF8,PSME3,PTPN11,RABGEF1,RBP1,RPL27,RPS6KA3,SF3B1,SIN3A,SLC2A3,SMAD2,STAM,TCP1,TF,TPT1,USP47,VCAM1,WTAP,XPO1	62
Cell Death and Survival	Neuronal cell death	7.47E-05	AARS1,APOE,ATP1A2,ATP2C1,CAMK2B,CIAPIN1,DPYSL3,EHD4,GAD2,GSTM5,HK2,HMOX2,HSPA5,HSPB1,IDE,KIF1A,LDHA,MEF2D,NGFR,NPM1,NQO2,PAFAH1B1,PARK7,PDCD6IP,PLRG1,PSME3,PTPN11,SLC25A12,SMPD2,STAM,TCP1,TF,WFS1	33
Cell Death and Survival	Cell viability	1.13E-04	AGO2,ALB,ANXA5,APOE,ATP1A2,ATP2C1,CAMK2B,CAPN2,CAV1,CBR1,CCAR1,CIAPIN1,DHX9,DNAJB6,EHD4,EZR,FLNA,GAD2,GPR37,HK2,HMGN1,HMOX2,HSPA1A/HSPA1B,HSPA5,HSPB1,IDE,IK,KDM1A,KIF1A,LDHA,MVP,MYO18A,NDUFA13,NGFR,NUP155,PARK7,PKM,PLRG1,POR,PPFIA2,PRPF8,PSME3,PTPN11,RABGEF1,RBP1,RPL27,RPS6KA3,SF3B1,SIN3A,SLC2A3,SMAD2,STAM,TCP1,TF,USP47,VCAM1,WTAP,XPO1	58
Cell Death and Survival	Cell death of neuroblastoma cell lines	1.52E-04	APOE,CAPN2,DHX9,GPR37,HSPA1A/HSPA1B,HSPA5,IDE,MARCHF5,MEF2D,NGFR,PARK7,TCP1,WDR5	13
Cell Death and Survival	Cell death of tumor cell lines	1.97E-04	AKAP12,ALB,ANXA5,AP2M1,APOE,ARHGDIA,ATP2A2,BBS4,CAPN2,CAV1,CBR1,CCAR1,CLTC,COX7A2L,CTBP2,DDX3X,DHODH,DHX9,DIDO1,DYNC1H1,EXOG,EZR,GPR37,GSN,HCFC1,HDAC6,HK2,HSPA1A/HSPA1B,HSPA5,HSPB1,IDE,MAP2K2,MARCHF5,MEF2D,MVP,NDUFA13,NGFR,NPM1,NUDCD3,OGFOD1,PARK7,PDCD6IP,PHGDH,PKM,PTPN11,RPL27A,RPS6KA3,RTN4,SARNP,SLC3A2,SMAD2,SMPD2,SUPT6H,TAGLN2,TCP1,TF,TPD52,TPT1,TXN2,USP47,VCAM1,WDR5,XPO1	63
Cancer,Cell Death and Survival,Organismal Injury and Abnormalities ,Tumor Morphology	Cell death of cancer cells	2.03E-04	ANXA1,HSPA1A/HSPA1B,NGFR,NUP155,NUP54,NXF1,PRPF8,RPL27,RPL27A,RPL3,RPS12,RPS13,RPS16,RTN4,Sf3a2,SF3B1,Snrpg,TF,VCAM1	19

Cell Death and Survival,Skeletal and Muscular Disorders	Cell death of muscle cells	4.16E-04	ACSL1,APOA1,CAV1,DMD,GSN,HADHA,HK2,HMOX2,HSPB1,MEF2D,NDUFA13,NGFR,PARK7,PLRG1,PTPN11,RTN4,TPT1,WTAP	18
Cardiovascular Disease,Cell Death and Survival,Organismal Injury and Abnormalities,Skeletal and Muscular Disorders	Cell death of cardiomyocytes	4.96E-04	ACSL1,APOA1,GSN,HADHA,HK2,HMOX2,HSPB1,MEF2D,NDUFA13,PARK7,PLRG1,PTPN11,RTN4	13
Cell Death and Survival	Cell viability of tumor cell lines	5.22E-04	AGO2,ALB,APOE,ATP1A2,ATP2C1,CAMK2B,CAPN2,CAV1,CBR1,CCAR1,DHX9,DNAJB6,FLNA,HK2,HSPA1A/HSPA1B,HSPA5,HSPB1,IK,KDM1A,KIF1A,LDHA,MYO18A,NDUFA13,NGFR,NUP155,PARK7,PKM,PPFIA2,PRPF8,PTPN11,RPL27,RPS6KA3,SF3B1,SIN3A,SLC2A3,SMAD2,TCP1,TF,USP47,XPO1	40
Cell Death and Survival	Cell death of nervous tissue cell lines	9.33E-04	HDAC6,HSP90AA1,MEF2D,NGFR,NPM1,PARK7,SMPD2	7
Cancer,Cell Death and Survival,Organismal Injury and Abnormalities,Tumor Morphology	Cell death of tumor cells	1.18E-03	ALB,ANXA1,CAV1,HSPA1A/HSPA1B,NGFR,NUP155,NUP54,NXF1,PRPF8,RPL27,RPL27A,RPL3,RPS12,RPS13,RPS16,RTN4,Sf3a2,SF3B1,Snrpg,TF,VCAM1	21
Cell Death and Survival,	Apoptosis of tumor cell lines	1.48E-03	AKAP12,ALB,ANXA5,AP2M1,APOE,ARHGDIA,BBS4,CAPN2,CAV1,CCAR1,CLTC,COX7A2L,CTBP2,DHODH,DHX9,DIDO1,DYNC1H1,EXOG,EZR,GSN,HCFC1,HDAC6,HK2,HSPA1A/HSPA1B,HSPA5,HSPB1,IDE,MARCHF5,MVP,NDUFA13,NGFR,NPM1,OGFOD1,PAR	49

			K7,PDCD6IP,PHGDH,PKM,PTPN11,RPS6KA3,RTN4,SMAD2,TAGLN2,TCP1,TF,TPD52,TPT1,TXN2,WDR5,XPO1	
Cell Death and Survival	Apoptosis of retinal cells	1.60E-03	HSPA5,HSPB1,KIF3A,NGFR,PTPN11	5
Cell Death and Survival	Apoptosis of neuroblastoma cell lines	1.60E-03	APOE,CAPN2,DHX9,HSPA1A/HSPA1B,HSPA5,IDE,NGFR,WDR5	8
Cell Death and Survival	Cellular degradation	1.83E-03	ALG10,ANK3,APOE,CAV1,CRYAA/CRYAA2,DPYSL3,DYNC1H1,Folh1,GPM6B,GPR37,HSPA1A/HSPA1B,KIF1A,NGFR,PARK7	14
Cell Death and Survival	Cell viability of leukemia cell lines	2.24E-03	CBR1,CCAR1,DNAJB6,HSPA5,NUP155,PTPN11,TCP1	7
Cell Death and Survival,Organismal Injury and Abnormalities,Renal and Urological Disease	Cell death of kidney cells	2.37E-03	AAK1,ALB,APOE,ATP2B2,EZR,GPR37,GSN,GSTM5,HDAC6,HK2,HSPA1A/HSPA1B,HSPA5,HSPB1,LDHA,NGFR,PARK7,PDCD6IP	17
